# Recurrent Thyrotoxicosis due to Both Graves' Disease and Hashimoto's Thyroiditis in the Same Three Patients

**DOI:** 10.1155/2016/6210493

**Published:** 2016-05-31

**Authors:** Ashley Schaffer, Vidya Puthenpura, Ian Marshall

**Affiliations:** Department of Pediatrics, Rutgers-Robert Wood Johnson Medical School, 89 French Street, New Brunswick, NJ 08901, USA

## Abstract

Hashimoto's thyroiditis (HT) and Graves' disease (GD) are the 2 most common autoimmune disease processes affecting the thyroid gland. The relationship between the two is complex and not clearly understood. It has been theorized that HT and GD are 2 separate disease processes due to unique genetic differences demonstrated by genome studies. On the other hand, based on occurrence of both HT and GD in monozygotic twins and within the same family, they have been regarded to represent 2 ends of the same spectrum. This case report describes 3 patients who presented with thyrotoxicosis due to both GD and HT. The initial presentation was thyrotoxicosis due to GD treated with antithyroid medication followed by temporary resolution. They all subsequently experienced recurrence of thyrotoxicosis in the form of Hashitoxicosis due to HT, and then eventually all developed thyrotoxicosis due to GD, requiring radioablation therapy.

## 1. Introduction

Hashimoto's thyroiditis (HT) and Graves' disease (GD) are 2 autoimmune thyroid diseases that account for the majority of acquired thyroid dysfunction in the pediatric population [1, 2]. It has been suggested that they are 2 entirely separate disease processes due to unique genetic differences demonstrated by genome studies [3]. On the other hand, based on occurrence of both HT and GD in monozygotic twins [4, 5] and in the same family [6, 7], they have been regarded to represent 2 ends of the same spectrum. A common mechanism proposed for their development is loss of tolerance to multiple thyroid antigens, including TSH receptor (TSHR), thyroglobulin, and thyroid peroxidase [8]. This leads to T lymphocyte infiltration of the thyroid gland [9] that can then follow 2 separate pathways, depending on the balance between T-helper 1 (Th1) and T-helper 2 (Th2) cells. Th1-cell-mediated autoimmunity leads to thyroid cell apoptosis and hypothyroidism in HT while a hyperreactive Th2-mediated humoral response against TSHR with stimulatory antibodies results in GD thyrotoxicosis [10, 11].

Although the exact incidence of HT in the pediatric population is unknown, it is much more frequent than GD [12]. As the presentation is usually asymptomatic, the diagnosis is commonly made incidentally by routine biochemical testing [13]. Clinically, HT can present with a firm, nontender goiter and occasionally with clinical evidence of hypothyroidism [13]. Rarely, HT can present with Hashitoxicosis, which is a transient form of thyrotoxicosis that results from release of preformed thyroid hormone due to inflammatory destruction of thyroid cells [14]. As inflammation resolves and because thyroid hormone release is not due to ongoing stimulation of TSHR, resolution typically occurs within a few months. It is usually asymptomatic, with typically only mild clinical symptoms of thyrotoxicosis if present [15].

Although GD is much less frequent than HT, with an incidence of about 1 : 10,000, it is the most common cause of thyrotoxicosis in the pediatric population [16]. Clinically, GD can present with a firm, nontender goiter, ophthalmopathy, a peripheral tremor, tongue fasciculations, tachycardia, and/or hypertension [1].

Diagnosis of HT is confirmed by presence of anti-thyroid peroxidase antibodies (anti-TPO Ab) and anti-thyroglobulin antibodies (anti-TG Ab) [17]. Diagnostic testing for GD relies on identification of TSHR autoantibodies that are measured by 2 different assays. The first is a radioreceptor assay that measures the ability of TSHR autoantibodies to compete with radiolabeled thyroid stimulating hormone (TSH) to bind to TSHR. These are commonly referred to as TSH binding inhibitor immunoglobulins (TBII) [18]. The second diagnostic test is a bioassay that measures the ability of TSHR autoantibodies to stimulate TSHR activity via cyclic adenosine monophosphate (cAMP) production [18]. These antibodies, which are known as thyroid stimulating immunoglobulins (TSIG), are the direct cause of thyrotoxicosis in GD.

Interestingly, anti-TPO Ab and anti-TG Ab can be detected in up to 70% of patients with GD, in addition to TBII and TSIG antibodies at the time of diagnosis [19]. However, the converse is not true in HT, where only TPO and/or TG antibodies are typically elevated [19].

We report 3 patients who presented with biochemical and clinical thyrotoxicosis due to GD and then after presumed spontaneous resolution of initial thyrotoxicosis experienced recurrence of biochemical thyrotoxicosis due to Hashitoxicosis, followed by a third period of biochemical and clinical thyrotoxicosis due to GD.

## 2. Case Presentation

*Case 1*. A 15-year-old female was diagnosed with thyrotoxicosis based on elevated free T4 (FT4) of 2.4 ng/dL (0.9–1.4) and suppressed TSH of 0.02 mIU/L (0.5–4.3) identified in work-up for irregular menses. Additional testing demonstrated elevated anti-TPO Ab at 180 IU/mL (0–35) and anti-TG Ab at 136 IU/mL (0–20); TBII were elevated at 22% (≤16), with TSIG within the normal range at 119% (≤125). Physical examination revealed a firm, nontender goiter only. I^123^ thyroid uptake and scan revealed increased 4-hour uptake at 34% (5–15%) and 24-hour uptake at 62% (15–35%).

Thyrotoxicosis due to GD was diagnosed but not treated due to absence of significant symptoms. After 6 months, worsening biochemical thyrotoxicosis associated with palpitations, insomnia, loss of weight, tongue fasciculations, peripheral tremor, tachycardia, and hypertension developed. Testing showed peak FT4 of 10.4 ng/dL and suppressed TSH of 0.01 mIU/L. TBII antibodies had increased to 49% with TSIG positive at 158%. Methimazole (MMI) therapy was started, with biochemical and clinical resolution of thyrotoxicosis within 2 months. After 18 months on therapy, with GD antibodies negative, MMI was discontinued to assess spontaneous resolution. She remained biochemically and clinically euthyroid for 4 months off MMI. Biochemical thyrotoxicosis without clinical symptoms developed after 4 months (peak FT4 of 2.4 ng/dL and TSH of 0.01 mIU/mL) with repeat anti-TPO and TG antibody levels at >1000 IU/mL and 147 IU/mL, respectively, and TBII and TSIG remaining negative. Repeat I^123^ thyroid uptake and scan revealed low 4-hour uptake of 2.5% and low 24-hour I^123^ uptake of 2.3%. This presentation was consistent with Hashitoxicosis, and because of mild nature and anticipation of its transient course antithyroid therapy was not initiated.

After 6 weeks, primary hypothyroidism actually developed (FT4 of 0.6 ng/dL and TSH of 25.66 mIU/mL) for which thyroxine replacement therapy was started. However, within 3 months, clinical and biochemical thyrotoxicosis was diagnosed which, despite discontinuation of therapy, deteriorated (peak FT4 of 3.9 ng/dL and TSH of 0.01 mIU/mL). Repeat I^123^ thyroid uptake and scan revealed elevated 4-hour and 24-hour uptake at 34% and 62%, respectively. ^131^I radioiodine ablation (RAI) was successfully performed with development of primary hypothyroidism within 2 months when thyroxine replacement therapy was restarted.

*Case 2*. A 14-year-old male presented with a 2-month history of palpations, jitteriness, insomnia, heat intolerance, and 10 lb weight loss. Initial examination revealed a nontender, firm goiter, tongue fasciculations, peripheral tremor, increased deep tendon reflexes, tachycardia, and hypertension. He was diagnosed with thyrotoxicosis due to GD based on wFT4 of 5.6 ng/dL and TSH of <0.01 mIU/mL and positive TBII at 34% (≤16) and TSIG at 130% (≤125); anti-TPO Ab and anti-TG Ab were positive at 107 IU/mL (<35) and 90 IU/mL (<20), respectively. He was treated with MMI therapy, which was then discontinued after 24 months, after which he remained clinically and biochemically euthyroid for a 12-month period.

Although asymptomatic, follow-up testing revealed biochemical thyrotoxicosis (peak FT4 of 3.9 ng/dL and TSH of 0.01 mIU/mL), with anti-TPO and anti-TG Ab levels at 308 IU/mL and 147 IU/mL, respectively, and negative TBII and TSIG antibody levels. I^123^ thyroid uptake and scan demonstrated low 4-hour uptake of 3% and 24-hour uptake of 5%. Hashitoxicosis was then diagnosed but did not require treatment. However, subsequent clinical and biochemical monitoring revealed increasing FT4 levels with associated development of clinical thyrotoxicosis. Repeat I^123^ thyroid uptake and scan demonstrated elevated 4-hour and 24-hour uptakes at 70% and 82%, respectively. He underwent RAI with development of hypothyroidism within 1 month for which he has been on thyroxine replacement therapy.

*Case 3*. The fraternal twin sister of Case 1 presented at 17 years of age to our emergency room with jitteriness, anxiety, tongue fasciculations, peripheral tremor, and hypertension and tachycardia. Testing showed extremely elevated FT4 at >7.77 ng/dL (0.9–1.8) with TSH suppressed at 0.01 mIU/mL (0.35–5.5); TSIG was positive at 432% (<140) and TBII at 83.2% (≤16), with positive anti-TPO Ab at 606 IU/mL (<35); anti-TG Ab was negative. She was diagnosed with thyrotoxicosis due to GD for which she was started on MMI therapy. After therapy for 18 months and with negative TSIG and TBII antibodies, a trial off MMI was initiated. She remained clinically and biochemically euthyroid off MMI for a period of 12 months at which time biochemical thyrotoxicosis developed. FT4 peaked at 3.0 ng/dL with TSH suppressed at 0.002 mIU/mL. Anti-TPO Ab remained positive at 612 IU/mL with TSIG and TBII levels still negative. She remained asymptomatic. I^123^ thyroid uptake and scan revealed low 4-hour uptake of 2.9% (5–15) and low 24-hour uptake of 4.7% (10–35), suggestive of Hashitoxicosis. Subsequently, she developed clinical signs of thyrotoxicosis with peak FT4 of 7.4 ng/dL and suppressed TSH at 0.001 mIU/mL. TSIG and TBII were now positive at 506% and 78.3%, respectively, with anti-TPO Ab positive at >900 IU/mL. MMI was restarted for recurrence of thyrotoxicosis due to GD followed by RAI after her repeat I^123^ thyroid uptake and scan revealed elevated 4-hour and 24-hour uptakes of 66 and 68%, respectively. Subsequent to RAI, she developed primary hypothyroidism that was treated with thyroxine replacement therapy.

## 3. Discussion

These are three very interesting patients who presented with 3 phases of thyrotoxicosis, initially with both biochemical and clinical thyrotoxicosis due to GD, followed by off MMI therapy by recurrence of biochemical thyrotoxicosis only due to Hashitoxicosis, and then again with both biochemical and clinical thyrotoxicosis due to GD.

The exact relationship between HT and GD continues to be debated. They have been suggested to be 2 separate disease processes partly based on whole-genome scanning studies in humans that revealed unique differences between loci associated with HT and GD [3]. Alternatively, they have been regarded as 2 ends of the same spectrum. This is based on reports that describe the occurrence of HT in one and GD in the second of monozygotic twins [4, 5, 20], the occurrence of HT and GD in the same family [6], and HT following GD in the same patient [21].

It cannot be argued that Hashitoxicosis and not GD was the cause of the initial thyrotoxicosis in all 3 patients. Based on the severity of thyrotoxicosis, presence of clinical symptoms and signs, need for pharmacological therapy, duration of thyrotoxicosis, and presence of positive TSIG and TBII antibodies, it is reasonable to conclude that the etiology of the initial thyrotoxicosis was GD.

The recurrence of thyrotoxicosis, associated with presence of HT antibodies when GD antibodies remained negative, and mild course associated with absence of clinical symptoms and signs were all suggestive of Hashitoxicosis and not GD. Furthermore, repeat I^123^ uptake and scans revealed uptake indicative of an inflammatory thyroiditis associated with HT and not increased uptake diagnostic for GD.

Another possible but unlikely explanation, at least for the transition from thyrotoxicosis to eventual hypothyroidism in the patients, could have been occurrence of TSHR autoantibodies in GD that inhibit TSH binding to TSHR (TSHR blocking antibodies or TSH stimulation blocking immunoglobulins) with subsequent hypothyroidism [18]. However, not only is presence of these antibodies an extremely rare biochemical phenomenon, but also negative TBII testing at that time suggested absence of these and other TSHR autoantibodies.

We believe this report is important as not only is it the first to report thyrotoxicosis due to GD, then due to Hashitoxicosis, and then due to GD in the same individuals, but also the cooccurrence of these 2 autoimmune processes highlights the concept that these are not separate processes but parts of the same autoimmune spectrum.
